# Dominance of Particulate Mercury in Stream Transport and Rapid Watershed Recovery from Wildfires in Northern California, USA

**DOI:** 10.1021/acs.est.4c09364

**Published:** 2024-12-04

**Authors:** Peijia Ku, Martin Tsz-Ki Tsui, Habibullah Uzun, Huan Chen, Randy A. Dahlgren, Tham C. Hoang, Tanju Karanfil, Huan Zhong, Ai-Jun Miao, Ke Pan, James S. Coleman, Alex Tat-Shing Chow

**Affiliations:** † Department of Biology, University of North Carolina at Greensboro, Greensboro, North Carolina 27402, United States; ‡ Environmental Sciences Division, Oak Ridge National Laboratory, Oak Ridge, Tennessee 37831, United States; § School of Life Sciences, The Chinese University of Hong Kong, Shatin, N.T., Hong Kong SAR, China; ∥ Department of Earth and Environmental Sciences, The Chinese University of Hong Kong, Shatin, N.T., Hong Kong SAR (852) 3943-5433, China; ⊥ State Key Laboratory of Marine Pollution, City University of Hong Kong, Kowloon Tong, Kowloon, Hong Kong SAR, China; # Department of Environmental Engineering, Istanbul Technical University, Istanbul 34467, Turkey; ∇ Department of Environmental Engineering and Science, Clemson University, Clemson, South Carolina 29625, United States; ○ Department of Land, Air and Water Resources, University of California, Davis, California 95616, United States; ◆ School of Fisheries, Aquaculture and Aquatic Sciences, Auburn University, Auburn, Alabama 36849, United States; ¶ School of the Environment, State Key Laboratory of Pollution Control and Resource Reuse, Nanjing University, Nanjing 210023, China; †† Shenzhen Key Laboratory of Marine Microbiome Engineering, Institute for Advanced Study, Shenzhen University, Shenzhen 518060, China

**Keywords:** mercury, methylmercury, fluvial transport, wildfire, rainstorms

## Abstract

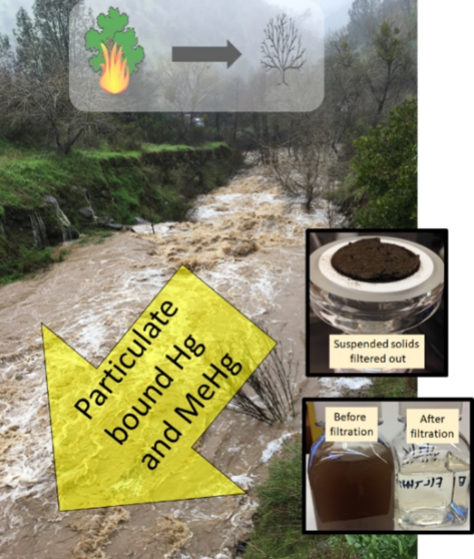

Frequency and intensity of wildfires are expected to increase due to climate change, especially in areas with a long summer drought. Forests are a major sink for the global pollutant mercury (Hg), and fluvial transport of Hg from recently burned watersheds has not been widely investigated. Here, we examined two years of fluvial transport of Hg and its speciation (total Hg, methyl-Hg, particulate, and dissolved forms) under storm events and baseflow in two recently burned watersheds with different burned proportions and one nonburned reference watershed in the Coastal Ranges of northern California. We examined postfire storm-event transport of Hg and its methylated form (methyl-Hg), addressed the importance of the “initial runoff pulse” to postfire Hg fluvial transport and its predominant association with suspended solids, and elucidated potential sources of Hg exports from the burned landscapes using geochemical indicators, which suggested that ash materials were likely the significant sources of particulates in the first high-flow season postfire but not subsequently. The maximum total suspended solid and total Hg levels in the “first pulse” at the severely burned watershed were 442 and 46 times higher, respectively, than those at the reference watershed. Stream suspended solid and Hg levels declined substantially in the burned watersheds after just a few months of rainfall likely due to the rapid regrowth of vegetation commonly observed in postfire landscapes, implying that the wildfire effects on immediate Hg inputs from the burned landscape are at most transient in nature.

## Abstract 2

The postfire “initial storm flush” led to elevated mercury and methylmercury exports in water from burned watersheds, with the majority of the mercury being associated with suspended particulate matter. Both particulate and dissolved mercury levels declined drastically after a few months of rainfall/runoff likely due to the rapid regeneration of vegetation in the watersheds which is common in postfire vegetated landscapes.

## Introduction

A perilous consequence of global climate change is an increased frequency and intensity of wildfires, which is especially evident in forested landscapes with prolonged dry summers, such as the western United States.^1^ Vegetation burned by wildfire can physically and chemically alter the properties and structure of surface soils and the destructively high temperatures (up to 1000 °C) can lead to increased water repellency, reduced infiltration rates, and ultimately higher surface runoff and erosion.^2^ After wildfires, the burned materials (i.e., ash layer) remaining on the soil surface can be wind-blown and/or water-eroded, and transported laterally by runoff.^3,4^ The fluvial transport of ash-laden materials represents a potential threat to downstream aquatic environments since the runoff often contains a mixture of organic and ash debris, nutrients to fuel eutrophication (i.e., nitrogen and phosphorus), persistent organic contaminants (e.g., polyaromatic hydrocarbon), and toxic heavy metals (e.g., lead and mercury).^2−8^

Through dry and wet deposition, forests represent a dominant sink for atmospheric mercury (Hg),^9^ but wildfires can drastically alter Hg cycling in forests. For example, the majority of Hg sequestered in foliage, litter, and surface debris is volatilized as gaseous Hg(0).^10,11^ Despite extensive volatilization by wildfires, a substantial amount of Hg [mainly as inorganic Hg or Hg(II)] remains in the burned landscape in soils and wildfire ash, and the chemical form, reactivity, and bioavailability of the remaining Hg can be highly altered during the intensive and prolonged burning process.^6,12,13^ Our recent study in northern California (USA) showed that Hg in black ash (after moderate-temperature burning) contained a higher proportion of Hg in a recalcitrant form than Hg in unburned leaf litter, implying a different environmental fate of Hg associated with these burned residuals in postfire landscapes.^6^

While wildfire causes extensive environmental damage during the burning period, a large concern exists during the subsequent rainy periods due to the high propensity for water erosion and contamination of downstream aquatic ecosystems, risking the resident food webs and the quality of source water for drinking.^14^ While a few studies have documented changes to Hg levels postfire,^5,15−17^ only Kelly et al.^16^ measured unfiltered total Hg (THg) and its neurotoxic form, methylmercury (MeHg), immediately postfire, and a recent study by Baldwin et al.^17^ measured unfiltered and filtered THg and MeHg in streams after almost a year postfire. Ultimately, any increases in MeHg, if occurring, may pose a greater biological risk, due to its capability for bioaccumulation and biomagnification in the natural food webs.^18,19^

In principle, forests (e.g., foliage, litter, and soil) contain the majority of Hg (>99%) in the form of inorganic Hg(II),^9,20^ while only trace amounts of MeHg generally exist in various forest compartments including vegetation, litter, and soils.^21−23^ However, it is not yet clear whether intensive heating from wildfire can break down MeHg in the residual materials. Notably, our preliminary analysis of black ash previously collected in northern California (with only THg reported in Ku et al.^6^) showed that the ash contained measurable, but very low amounts of MeHg (mean ± S.D.: 0.03 ± 0.01 ng/g dry wt.; *n* = 3) compared to their total Hg concentrations (11.3 ± 5.3 ng/g dry wt.; *n* = 3), with the mean percentage of MeHg as THg (i.e., %MeHg) being only 0.27%. Thus, a small amount of MeHg inherent in the residual ash and soil layers after wildfire may still be readily available for downstream transport and deposition following runoff/erosion.^14^ Upon deposition of the residues and soils into the downstream surface sediments, the deposited Hg(II) may be microbially converted (e.g., sulfate-reducing bacteria)^24^ to MeHg in the downstream sediment^8^ when the geochemical conditions become favorable.^25^ We posit that the fluvial transport of “native” MeHg would be rapid without the need of in situ production and can be captured by studying the fluvial transport in streams, whereas the in situ methylation of deposited Hg(II) in downstream sediments would proceed with a lag time that would largely depend on the in situ environmental conditions (e.g., sulfate availability for microbial sulfate-reduction and development of anoxic conditions) conducive to microbial MeHg production.^25^

In fluvial systems, Hg(II) and MeHg can be transported in the dissolved phase (e.g., < 0.45-μm) or in the particulate phase (e.g., ≥ 0.45-μm). In the dissolved phase, dissolved organic matter (DOM) is the predominant vector for Hg transport due to the strong affinity between Hg and the thiol group as an aqueous complex.^26^ Hence, many freshwater systems demonstrate a positive relationship between dissolved organic carbon (DOC, a common proxy for DOM) and Hg(II) or MeHg concentrations.^27−29^ In the particulate phase, it has been demonstrated that total suspended solids (TSS), a commonly measured water quality parameter, can account for the majority of variations in Hg(II) and MeHg concentrations of surface waters with high solid loading (e.g., agricultural fields).^30^ The importance of the dissolved vs particulate phase transport of Hg largely depends on the land-use types in the watershed (e.g., forest vs agriculture), perturbations in the landscape (e.g., fire, wood harvesting), and storm/runoff dynamics (e.g., antecedent conditions, rainstorm intensity/duration, soil infiltration, and sediment transport and type of sediment).^31^

In this study, we followed fluvial Hg transport dynamics in two watersheds impacted by destructive wildfires in the summer of 2015 in northern California (USA) and compared the results to a nearby nonburned reference watershed. The study watersheds occur in a region having relatively high background Hg concentrations in soils and rocks, largely existing as cinnabar, metacinnabar, and montroydite.^32^ In one watershed, >90% of the watershed area was burned (Cold Creek Watershed, impacted by Wragg Fire) while in another larger watershed only ∼15% of the watershed area was burned (Cache Creek Watershed, impacted by Rocky Fire).^6^ We aimed to (i) provide a complete storm-event/baseflow data set of Hg concentrations and speciation (i.e., THg, MeHg, both in particulate and dissolved forms) in streamflow for a two-year postfire period, especially addressing the importance of the “initial flush” in postfire fluvial Hg transport; (ii) investigate the relationships between particulate and dissolved THg or MeHg transport with variations in TSS and DOC, respectively; (iii) examine the temporal chemical signatures of suspended particulate matter by employing calcium (Ca) as demonstrated in our previous wildfire ash study^6^ to track the potential sources of Hg export from the burned landscapes; and (iv) evaluate the recovery of the burned watersheds with respect to Hg and MeHg fluvial transport in wildfire-prone northern California, an area expected to have more frequent and destructive wildfires under a warming climate.^33^

## Materials and Methods

### Study Sites and Sample Collection

**Figure 1:**
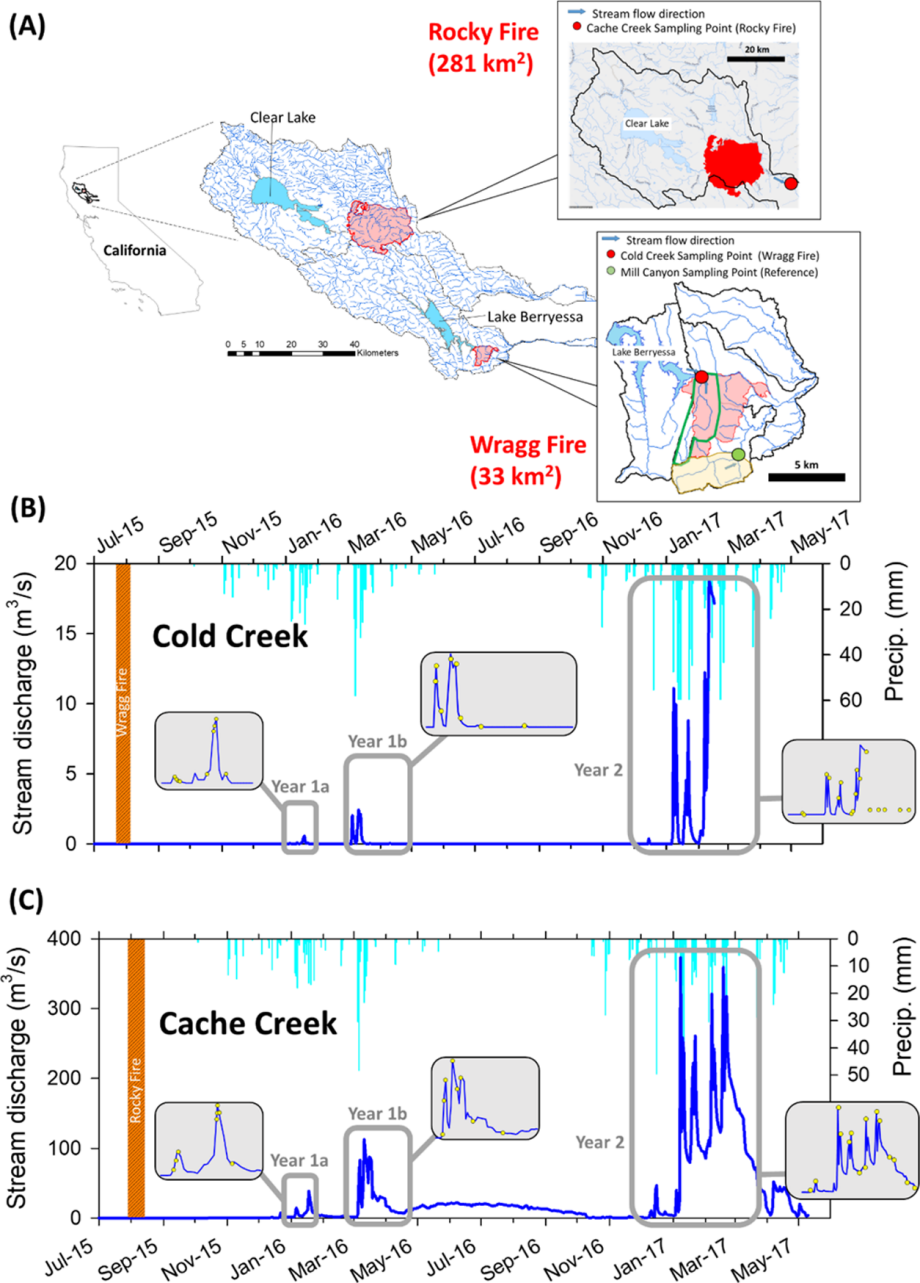
(A) Watershed locations in northern California and streamwater sampling points (shown by the green zigzag arrows) at Mill Canyon Creek (Reference), Cache Creek (Rocky Fire), and Cold Creek (Wragg Fire). The red-shaded areas and perimeters indicate the extent of the burned areas. For Cold Creek Watershed impacted by Wragg Fire, the green line indicates the area where the water flow is directed toward the sampling point for collection. Stream discharge and regional precipitation (weather data recorded at the Sacramento International Airport) in (B) Cold Creek (Wragg Fire) and (C) Cache Creek (Rocky Fire). Note the sampling periods highlighted as Year 1a, Year 1b, and Year 2. In the inset diagrams in (B) and (C), the yellow circles indicate the actual sampling dates at both study sites along the hydrographs.

In this study, we examined postfire fluvial Hg transport in two different streams in northern California. Based on a previous study^34^ and our ground-level observations (e.g., duff thickness, ash accumulation and color, and darkened trees), the fires can be described as moderate severity. In summer 2015, the Wragg Fire (22 July to 5 August) burned 33 km^2^ within the Cold Creek Watershed (>90% watershed area burned) while several wildfires (collectively referred to Rocky Fire; 29 July to 14 August, 2015) occurred in the Cache Creek Watershed downstream of Clear Lake, and burned a total of 383 km^2^ (*note:* Rocky Fire burned 281 km^2^) accounting for ∼15% of this watershed area (Figure 1A). Prefire vegetation was similar among the study sites, mainly composing of a mixture of oak savanna and woodlands, chaparral, and annual grasslands, while soils were mainly Inceptisols and Alfisols.^7^ The watersheds had 15–45% of slope in topography, implying a high tendency for water erosion. The region has a Mediterranean climate with rainfall occurring mainly between late October and April. We expected that the rainfall patterns falling onto both burned watersheds as well as the reference watershed (Mill Canyon Creek Watershed which is adjacent to Cold Creek Watershed impacted by Wragg Fire) would be similar following the same storm events.^7^ Both burned watersheds we studied mainly have forests and/or grasslands, although the lower Cache Creek Watershed, for which we did not sample, has much higher agricultural activities.

Our sampling crew visited each burned area approximately one month after the fires but before any rainfall events to collect wildfire ash and surface soil samples (0–5 cm depth below the ash layer) at both the Wragg and Rocky fire sites.^6^ We also collected unburned soil samples from immediately outside the fire perimeter of the Wragg Fire to examine and compare them with the chemical characteristics of the burned materials within the fire perimeter. It should be noted that the chemical properties of ash materials (except MeHg) were previously published,^6^ but Hg content in both the soil and streamwater samples has not been previously reported.

We identified Mill Canyon Creek Watershed (21.5 km^2^) adjacent to the Cold Creek Watershed as a nonburned reference watershed due to their similarities in geology, soils, vegetation, and relief (Figure 1A). A detailed description of the study area including geology, topography, vegetation, climate, and stream discharge can be found in Uzun et al.^7^ For all three streams, we collected streamwater samples (*see* details in Supporting Information (SI) Text S1) for two consecutive winter rainy seasons following the 2015 summer wildfires in order to assess both short-term, first flush, and longer-term impacts of wildfire on fluvial Hg transport and watershed recovery.

It should be noted that there was no streamflow in either Cold Creek or Mill Canyon Creek (*data not shown*) prior to winter rainfall so we were able to target the “first flush” in early January 2016, referred to as the Year 1a period (Figure 1B). For Cache Creek, there was baseflow throughout the dry summer period due to upstream inputs from Clear Lake, but we were able to identify a distinct “first flush” after the summer wildfires (Figure 1C). Overall, our sampling crew conducted relatively intensive sampling in January 2016 (referred to as “Year 1a”), March-April 2016 (referred to as “Year 1b”), and December 2016 through April 2017 (referred to “Year 2”). Due to the much higher precipitation amount in the second year, Year 2 had a significantly larger streamflow than the combined Year 1a and 1b flows in both Cold Creek and Cache Creek (Figure 1B,C). On the sampling day, water samples from all sites (note: lower frequency for Mill Canyon Creek in Year 1) were collected within 3–4 h. Samples were collected in both years, with counts for Mill Canyon Creek (Year 1: *n* = 6; Year 2: *n* = 16), Cold Creek (*n* = 17 for Year 1 and 2), and Cache Creek (*n* = 16 for Year 1 and 2). The sampling frequency in the reference stream was lower than the other two streams due to the greater evapotranspiration and soil infiltration that delayed streamflow generation in the nonburned watershed (Figure 1B,C). In Cold Creek and Mill Canyon Creek, the discharge estimation was based on the nearby Putah Creek (Cold Creek/Mill Canyon Creek = Downstream gauge – Berryessa dam release), but on 17 February 2017, there was an ungauged overflow from Lake Berryessa due to the extremely high rainfall, and thus subsequent streamflow estimation was not possible for Cold Creek (Figure 1B).

### Sample Processing and Mercury Analyses

For all streamwater samples except those from the reference watershed, we filtered and analyzed both the unfiltered and filtered portions following our established laboratory protocols.^35−37^ We analyzed only unfiltered streamwater samples from the reference watershed for THg in Year 1. In selected samples with high solid concentrations, we obtained sufficient amounts of suspended particles to analyze their Hg and other geochemical contents directly. Streamwater samples were analyzed for THg and MeHg, whereas solid-phase samples (suspended particulates, ash, and soil) were analyzed for THg and Ca. A summary of sampling protocols is provided in SI Text S2, Hg analyses in SI Text S3, and other geochemical analyses in SI Text S4 and SI Table S1.

### Statistical Analyses

One-way ANOVA followed by a Tukey’s post hoc multiple comparison test was conducted to assess significant differences with a *p* value of 0.05. Pearson’s linear regression and Spearman’s pairwise correlation analyses were conducted using SigmaPlot 12.5.

## Results and Discussion

### Streamflow Dynamics and Pulses of Suspended Particulates

Hydrologic patterns were similar in Cold Creek and Cache Creek for the two study years, with much smaller flows in Year 1 than in Year 2 (Figure 1B,C). During Year 1a (January 2016), there were two “pulses” in both Cold Creek (up to 0.05 and 0.57 m^3^/s) and Cache Creek (up to 14.4 and 38.5 m^3^/s). After a prolonged dry period, the storm events became substantially larger in the Year 1b period (March-April, 2016) in which a double peak hydrograph was observed in Cold Creek (up to 2.4 m^3^/s) compared to a relatively larger hydrograph peak in Cache Creek (up to 112.7 m^3^/s) (Figure 1B). In Year 2, the total amount of precipitation was ∼68% higher compared to the same period in Year 1 (December to April) (i.e., increased from 362 to 608 mm). Thus, streamflow in both Cold Creek (up to 18.8 m^3^/s) and Cache Creek (up to 321.0 m^3^/s) was substantially higher in Year 2. There were multiple large pulses in Cold Creek along with continuously high flows between storm events in Cache Creek during Year 2 (Figure 1C).

**Figure 2:**
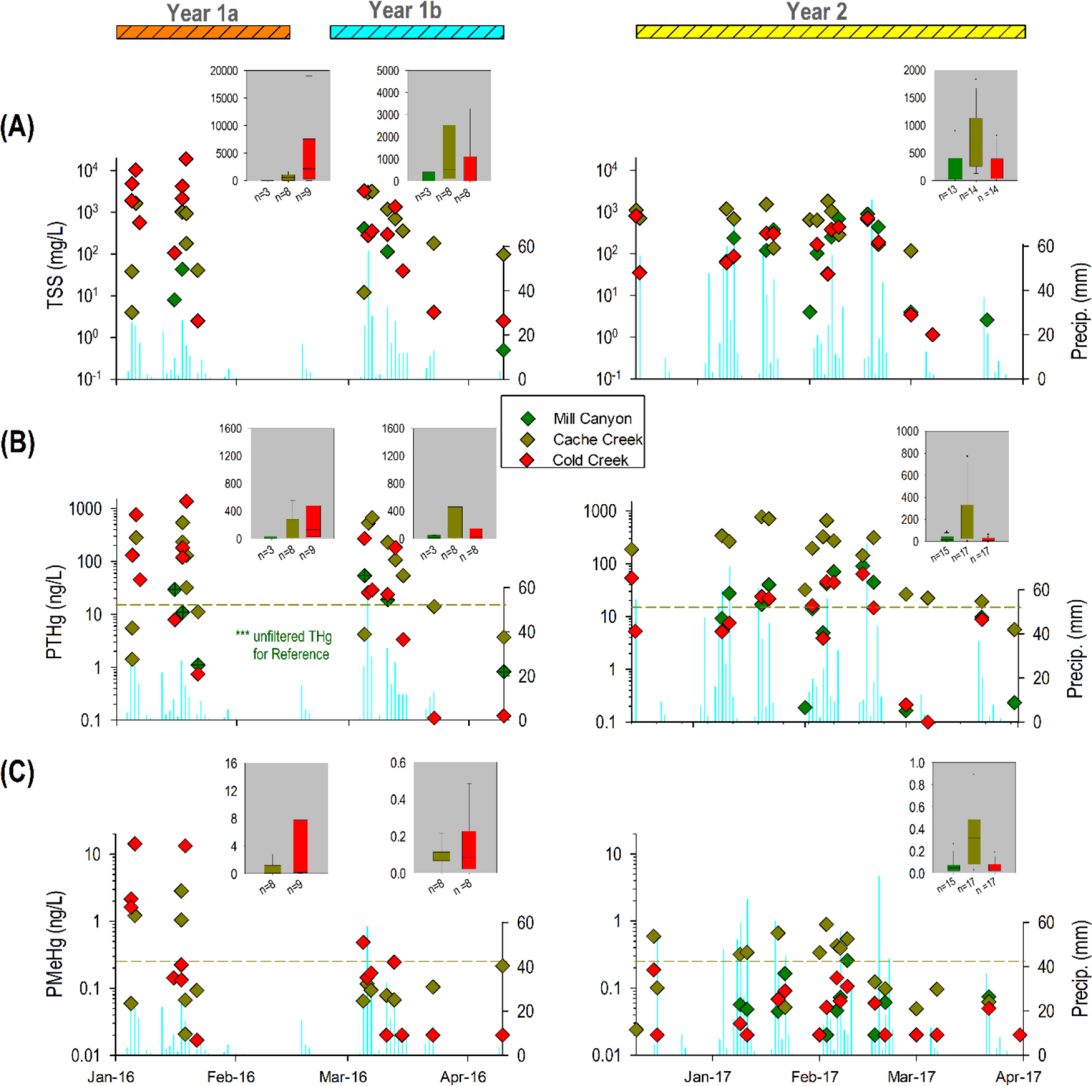
Temporal variation of (A) total suspended solids (TSS), (B) particulate total mercury (PTHg), and (C) particulate methylmercury (PMeHg) in streamwaters of the three study sites including Mill Canyon Creek (Reference), Cache Creek (Rocky Fire), and Cold Creek (Wragg Fire) during Year 1a, Year 1b, and Year 2. The dashed lines in (B) and (C) show the average PTHg and PMeHg levels recorded in a previous study at Cache Creek.^41^ ***Note that in (B) we only reported unfiltered THg vales for Reference site and the symbol would be denoted with a “cross” inside the symbol.

Overall, wildfire greatly elevated erosion and the levels of suspended solids (reflected by TSS measurements) in streamwater (*see* field and laboratory photos in SI Figure S1), with the highest level of TSS recorded (∼19 g/L) in Year 1a at Cold Creek (Figure 2A). Such extremely high TSS levels, despite transient in nature, were considerably higher compared to those reported in other postfire stream systems such as those recently recorded in southern California (up to 1.1 g/L)^38^ and far exceeded water quality criterion for surface water (e.g., < 0.1 g/L).^39^

In comparison, the reference watershed (Mill Canyon Creek) adjacent to Cold Creek had TSS levels 4 orders of magnitude lower during the same time period (up to 43 mg/L) (Figure 2A). However, the differences in TSS levels between Mill Canyon Creek and Cold Creek became smaller in Year 1b before becoming almost indistinguishable in Year 2 (*p* > 0.05) (Figure 2A). This implies a rapid decline in suspended sediment/erosion of this severely burned watershed within several months to less than a year. We attribute this phenomenon to the very rapid regrowth of vegetation upon rainfall owing to regeneration of stump sprouting shrubs/trees and germination/growth of annual grasses that quickly provided modest soil cover (*see* field photos at Cold Creek Watershed in SI Figure S2).^40^

In contrast, Cache Creek had much lower TSS levels during Year 1a, with the highest TSS level recorded at 1.6 g/L (Figure 2A), which was ∼1 order of magnitude lower than those observed in Cold Creek but similar to the recorded levels in southern California (up to 1.1 g/L).^38^ We attribute this large difference to the size differences of the watershed area and the proportion of the watershed area that was burned (i.e., 90% in Cold Creek vs. 15% in Cache Creek) (Figure 1A), which likely determines the landscape area exposed to elevated erosion and fluvial transport of suspended particulates. Interestingly, the TSS levels did not noticeably decline in Cache Creek over time, with median levels recorded at 561 mg/L (year 1a), 533 mg/L (year 1b), and 675 mg/L (year 2) (Figure 2A). Thus, the wildfire impact on TSS was not as apparent in Cache Creek as compared to Cold Creek, probably due to dilution from the large nonburned area (∼85%) in this larger watershed.

### Mobilization of Particulate Total Mercury

Due to the high amounts of suspended particulates and potentially different types of sediment in the initial storm-event pulses, the percentage of THg associated with suspended particulates varied widely over time in both Cold Creek and Cache Creek whereas it remained more consistent in Mill Canyon Creek (Figure 2B, SI Figure S3A and B). Overall, when THg levels in streamwater were elevated (e.g., >10 ng/L), the majority of THg (e.g., >60%) was associated with the suspended particulates in both Cold Creek and Cache Creek (SI Figure S3C and D). The particulate THg (PTHg) percentages were higher in Cold Creek (median: 84%) and Cache Creek (median: 96%) than Mill Canyon Creek (median: 69%). This implies that the major impact of wildfire is related to particulate matter potentially derived from burned landscapes and their associated Hg pools during rainstorm events. Further, the dissolved phase represents only a minor component of THg transported, and thus we first focus on the particulate phase of THg and MeHg as they represent the majority of Hg transported in the initial postfire period.

For PTHg, its temporal trend largely followed that of TSS in all three time periods in Cold Creek (Figure 2B), and similarly the temporal trend of PTHg in Cache Creek was nearly the same as TSS (Figure 2A). Even though TSS levels were much higher in Cold Creek during Year 1a, the median PTHg level in Cold Creek (131 ng/L) was about 1-fold higher than that in Cache Creek (81 ng/L) (Figure 2B). In the subsequent periods, PTHg was substantially reduced in Cold Creek (i.e., median PTHg: 25 ng/L in Year 1b and 12 ng/L in Year 2), which was opposite to the increasing median PTHg in Cache Creek (i.e., median PTHg: 81 ng/L in Year 1b and 196 ng/L in Year 2) (Figure 2B). Overall, PTHg in Cache Creek in Year 2 was much higher compared to the average values reported for the same watershed in a previous study.^41^ There were likely some sources of PTHg (regardless of whether wildfire-impacted or not) which would be mobilized by the much higher rainfall/erosion in Year 2 (Figure 2B), such as the Hg-polluted sediment from the upstream Sulfur Creek.^41^ Alternatively, it may be possible that the reduction of PTHg following wildfire in Cache Creek resulted from the volatilization loss of Hg during burning of forest biomass, but returned to greater soil erosion sources and/or enhanced soil-water repellence due to higher rainfall/erosion in Year 2.^33^

By examining the relationships between TSS and PTHg in both streams throughout the study periods (SI Figures S4A and S5A), it is clear that the THg content in the suspended particulates was consistently higher in Cache Creek than Cold Creek (i.e., the slope between TSS and PTHg was 201–334 ng/g vs 56–88 ng/g, respectively, while the calculated ratio of PTHg/TSS per individual sample ranged from 38 to 1,139 ng/g for Cache Creek and 9–298 ng/g for Cold Creek), corroborating that Cache Creek has inherently higher geologic background Hg concentrations,^41^ including cinnabar, metacinnabar, and montroydite in soils and rocks.^32^ Interestingly, the THg content of suspended particulates in Cold Creek decreased slightly in Year 2 (SI Figure S4A), suggesting that the wildfire ash and top soil preferentially eroded in Year 1 were enriched in Hg relative to the soil materials eroded in subsequent years. However, our soil THg measurements from Cold Creek Watershed did not show significant differences (*p* > 0.05) for THg in soils from unburned areas vs burned areas (collected under ash layer) (unburned area: 18.9–75.8 ng/g vs burned area: 5.9–111.7 ng/g) (SI Table S2), which may also suggest the severe burning may not appreciably influence the THg content of bulk surface soil. A previous study found that only the very top few centimeters of the soil layer experienced Hg loss after wildfire.^42^

The THg content of suspended particulates in Cache Creek was higher in Year 2 (Figure 2B), which may be attributed to the decreased export of wildfire ash which is typically lower in Hg content^6^ and increased transport of Hg-rich soil in the runoff. This increase may also result from a lag in sediment transport of burned materials through the much larger watershed or from a larger rainfall in Year 2. In Cold Creek, a distinct outlier (circled) occurred on 17 February 2017 (SI Figure S4A) that had very low THg content relative to the high TSS level. This occurred on the same day that the upstream Lake Berryessa began to spill excess water from its emergency spillway due to extremely large rainfall events. We speculate that extreme rainfall may have mobilized particulate matter from deeper soil horizons that had a lower Hg content than surface soils or ash materials not previously mobilized by smaller rainfall events (some ash materials also had very low THg levels; < 10 ng/g).^6^

Comparing across the three study periods in Cold Creek (SI Figure S4A), it can be inferred that the severe wildfire (∼90% of watershed) led to higher PTHg as related to elevated TSS levels in general, but the Hg content associated with the suspended particulates varied little overall (from 56 to 88 ng/g). In contrast, it seems that the less extensively burned Cache Creek Watershed (∼15% of watershed) was not appreciably impacted by wildfire as the THg content associated with the suspended particulates was only slightly elevated in Year 1a (233 ng/g) vs Year 1b (201 ng/g). However, the much higher rainfall in Year 2 likely mobilized some previously burned soil and/or some upstream soil and channel sediment deposits having higher Hg content (e.g., Sulfur Creek),^41^ resulting in an almost 50% increase in THg content on the suspended particulates (334 ng/g) (SI Figure S5A). Notably, we acknowledge that the PTHg-TSS ratios might be potentially influenced by the composition of suspended particles (i.e., the proportion of sand vs organic-rich) and changes in erosion patterns (e.g., due to increased rainfall or vegetation regrowth). However, due to limited field sample collection, we were unable to directly analyze the specific nature of these particles and the field hydrological paths.

Regarding Hg loads, in Year 1a, the daily Hg load peaked at 3,183 g/day with a median of 383 g/day in Cache Creek. This load increased to a peak of 8,716 g/day, with a median of 490 g/day in Year 1b, and further escalated to 18,356 g/day, with a median of 2,982 g/day in Year 2. These values are significantly higher than the previously reported Hg load of 213 g/day during storm events at Rumsey in 2000–2001,^41^ highlighting the impacts of a combination of wildfire and extreme rainfall events on Hg transport in Cache Creek. This trend suggests that as a larger watershed, Cache Creek experienced a substantial and sustained increase in Hg loads, indicative of prolonged transport of Hg-laden materials. In Cold Creek, the median load for Hg was 6.6 g/day and peaked at 675 g/day in Year 1a. The Hg load decreased (peaked at 107 g/day and median 9.7 g/day) in Year 1b, and further decreased (peaked at 54 g/day and median 1.9 g/day) in Year 2 even though rainfall was much higher in the second year. This trend suggests a recovery trajectory for Hg transport in Cold Creek as the effects of the wildfire diminished quickly over time. Comparatively, Mill Canyon Creek displayed significantly lower with a median 0.11 g/day in Year 1a, 3.23 g/day in Year 1b and 4.26 g/day in Year 2, demonstrating clearly the elevated Hg loads in Cold Creek due to destructive wildfire effects (compared to the effects of increased rainfall). These findings underscore the variability in watershed responses to wildfire and highlight the need to consider site-specific factors, such as climate, hydrology, vegetation recovery, and sediment dynamics, when evaluating postfire Hg transport.

### Identification of Origin of Suspended Particulates

**Figure 3:**
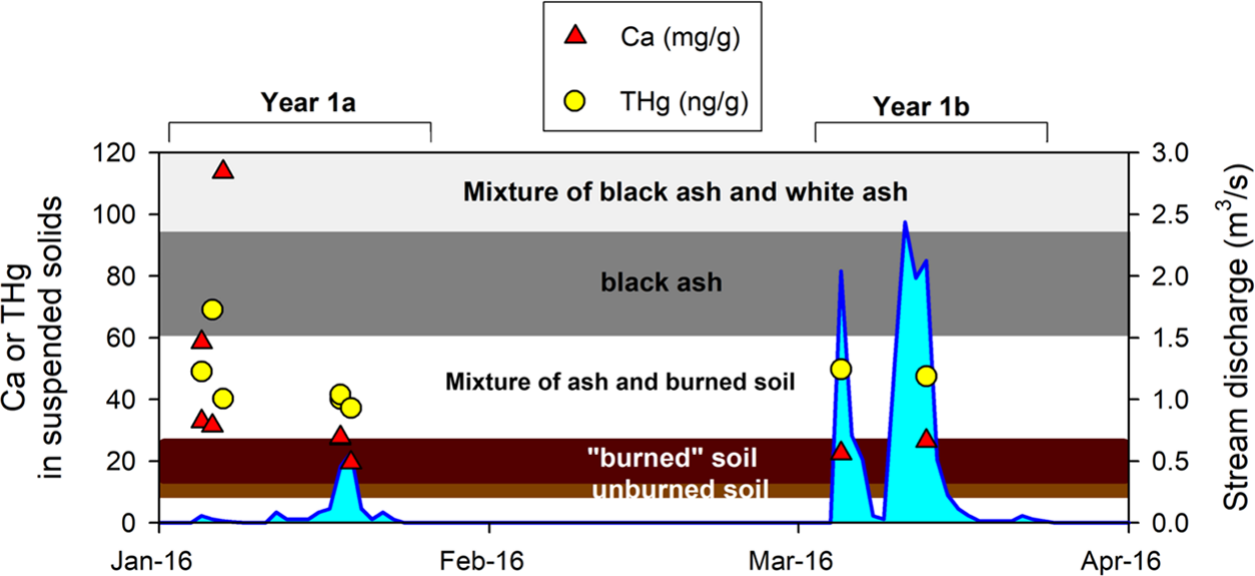
Temporal variations of calcium (Ca) and total Hg (THg) content in suspended solids in Cold Creek in Year 1a and Year 1b. Also shown are the range of Ca content determined for unburned soil outside fire perimeter of Wragg Fire, “burned” soil collected within the Wragg Fire perimeter, and black ash (low-severity) and white ash (high-severity) within Wragg Fire perimeter. The graph also shows the stream discharge (in blue-shaded areas) of Cold Creek for that period.

In Cold Creek, the very high TSS levels in Year 1a and Year 1b allowed us to collect enough suspended particulates to analyze them directly for THg and Ca contents. We also included black and white ash samples (previously reported in Ku et al.^6^), surface soils under black and white ash samples, and unburned soils collected outside the fire perimeter to assess wildfire effects on Hg concentrations. Based on the previous findings of Ku et al.,^6^ we employed Ca content as a robust geochemical indicator for distinguishing different potential sources of suspended particulates (Figure 3). Specifically, we found that early in Year 1a, a mixture of soil and ash represented the main sources of suspended particulates, but the proportion of ash quickly declined after 2–3 weeks, with the Ca content of the suspended particulates becoming essentially the same as those of the soils from the burned zone (Figure 3).

### Occurrence of Methylmercury in Streams after Wildfire

A primary concern following wildfires is whether it would elevate MeHg concentrations in downstream aquatic environments that could lead to increasing food web bioaccumulation.^14,16,17^ It is possible for MeHg to be derived directly from the terrestrial landscape or from Hg(II) deposited in sediments and subsequently methylated in downstream habitats. Our stream sampling protocols mainly capture the former pathway, i.e., the direct terrestrial inputs of MeHg formed before or after the wildfire (but before runoff). In Year 1a, the daily MeHg load peaked at 17.1 g/day with a median of 0.4 g/day in Cache Creek in Year 1a. The load showed a peak of 2.0 g/day in Year 1b with a median of 0.5 g/day and increased to 15.3 g/day with a median of 3.4 g/day in Year 2. These loads are similar to or higher than the previously reported MeHg load of 0.67–1.45 g/day during storm events at Rumsey, California in 2000–2001.^41^

Similar to Hg(II), the particulate phase was found as the dominant carrier of MeHg in streamwater. In Cold Creek, we found similar temporal patterns for TSS, PTHg, and PMeHg among the three study periods after the Wragg Fire (Figure 2). However, the temporal patterns were somewhat altered for Cache Creek in Year 1b when PMeHg did not follow close trends with TSS and PTHg (Figure 2). These PMeHg values were close to those of average values from a previous study at Cache Creek (*see* dashed lines in Figure 2C).^41^ Notably, the percentage of MeHg in the particulate phase represented 81–99% (median 88%) of MeHg in Year 1a and 83–96% (median 89%) in Year 1b, but decreased to 22–88% (median 46%) in Year 2 in Cold Creek, with the latter being comparable to those of the reference site in Year 2 (range from 55 to 88% and median 56%), suggesting the quickly diminishing effects from the burning on MeHg export to the stream after one year in Cold Creek. The percentage of MeHg in the particulate phase in Cache Creek was 28–96% (median 55%) in Year 1a, 45–84% (median 63%) in Year 1b, and 54–95% (median 91%) in Year 2, which would be likely driven by the increases in rainfall in Year 2. These values in Cache Creek were comparable to or slightly higher than those reported previously from samples collected between 2000 and 2001, with values between 22 and 71%.^41^

When we examined relationships between TSS and PMeHg (SI Figure S3B), there were significant positive relationships for Cold Creek among all three time periods. It appears that the regression slopes, which represent the average MeHg levels associated with the suspended particulates (0.1–0.8 ng/g), were indeed comparable to MeHg concentrations in surface sediment elsewhere (e.g., San Francisco Bay-Delta area, 0.72 ± 0.68 ng/g in Central Delta, 0.39 ± 0.19 ng/g in Prospect Slough, and 0.10 ± 0.10 ng/g in Cosumnes River).^43^ The range of values was also similar to MeHg concentrations measured in terrestrial landscapes, such as wildfire ash (*our preliminary results*), unburned surface soils, and vegetation elsewhere.^21−23^ This implies that MeHg was unlikely to be produced *in situ* during the short transient time of the particulate transport in well-aerated streams but rather derived directly from the terrestrial landscapes upon runoff/erosion. We also found that the MeHg content associated with suspended particulates declined substantially from Year 1a (median: 0.8 ng/g) to Year 1b (0.1 ng/g), which remained similar to that in Year 2 (0.2 ng/g), suggesting that the initial flush may be disproportionally more important for the mobilization of the PMeHg after wildfire (SI Figure S3B). One potential reason can be due to the higher MeHg content in the top organic horizon than the mineral soil underneath,^21^ and if completely not consumed by the wildfire, these partially burned organic soil layers can be mobilized by the initial flush.

In contrast, we found significant but weaker relationships between TSS and PMeHg in Cache Creek during the three study periods (SI Figure S5B). We attribute this discrepancy in part to mixing of different MeHg pools from burned (15%) and nonburned (85%) areas within this large watershed having high natural background levels of Hg.^41^ Notably, the slope of the regression indicated that the MeHg content associated with the suspended particulates in Cache Creek was actually similar to those in Cold Creek (SI Figure S5B) even though the THg content associated with suspended particulates was much higher in Cache Creek (SI Figure S5A). The MeHg levels found in suspended particulates in Cache Creek were found to be similar to those sediments reported previously in the downstream Cache Creek Nature Preserve and Cache Creek Settling Basin (0.2–0.7 ng/g).^44^

### Dissolved Pools of Organic Carbon and Mercury in Fire-Impacted Streams

While particulate pools are the dominant forms of THg and MeHg in both fire-impacted and nonimpacted systems, they play an especially critical role in postfire-impacted streams, where extensive ash and soil erosion driven by rainfall and runoff significantly increase particulate-bound mercury levels. However, the dissolved forms of Hg were possibly elevated through increased mobilization of DOM from these eroded materials having varying amounts of organic matter^7,45^ and a high pH that may promote DOM solubilization.^46^ Further, it is posited that dissolved forms of Hg(II) are more predisposed to methylation than particulate forms of Hg(II).^47^ Despite its lesser abundance, the dissolved/filtered pool of Hg(II) is still important to evaluate regarding its biogeochemical importance. Notably, the dissolved form of MeHg has been directly linked to its accumulation within lotic food webs,^18^ and thus any spike of dissolved pools of Hg(II)/MeHg in streams should not be simply ignored.

**Figure 4:**
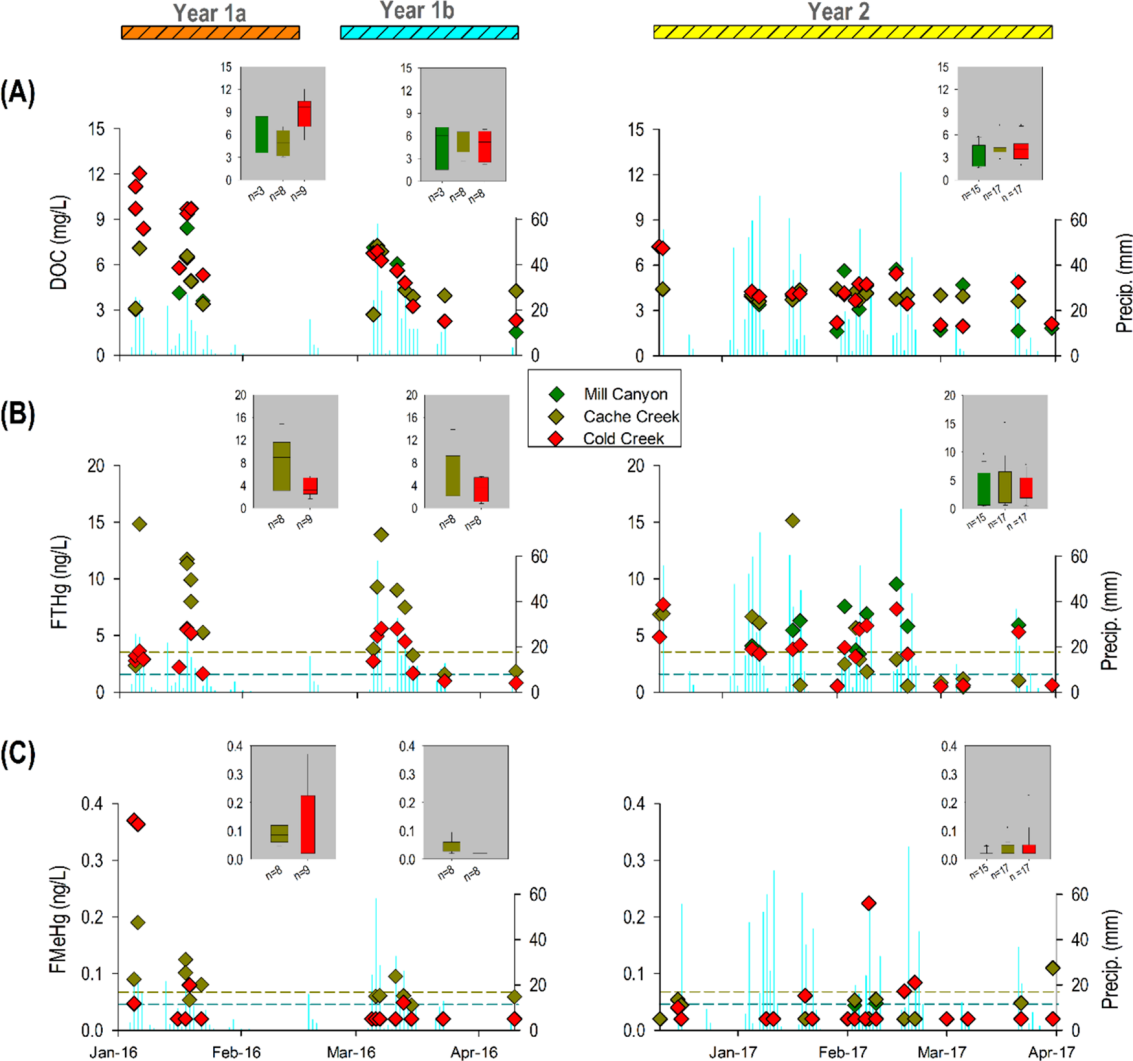
Temporal variation of (A) dissolved organic carbon (DOC), (B) filtered total mercury (FTHg), and (C) filtered methylmercury (FMeHg) in streamwaters of the three study sites including Mill Canyon Creek (Reference), Cache Creek (Rocky Fire), and Cold Creek (Wragg Fire) during Year 1a, Year 1b, and Year 2. The dark yellow dashed lines in (B) and (C) show the average FTHg and FMeHg levels recorded in a previous study at Cache Creek.,^41^ while the cyan dashed lines in (B) and (C) indicate the average FTHg and FMeHg levels previously recorded for a river in the coast range of northern California without Hg point sources.^53^

In Cold Creek, DOC levels were only slightly elevated (up to ∼12 mg/L) by the wildfire as opposed to the orders of magnitude increase in TSS (Figure 4A). Specifically, the median DOC in Year 1a was 9.7 mg/L, roughly double that of Year 1b (5.2 mg/L) and Year 2 (4.1 mg/L) (Figure 4A). FTHg varied in a temporal fashion similar to that of DOC; however, the median FTHg did not change much over the three study periods (Figure 4B). Following prolonged periods with low flow (i.e., baseflow dominated by groundwater inputs), both DOC and FTHg were distinctly elevated during subsequent storm events, with FTHg showing higher values than the average values as inferred from other studies (*see* dashed lines in Figure 4B). We observed relatively higher FMeHg concentrations (0.3–0.4 ng/L) during Year 1a, but the peak of FMeHg did not coincide with that of FTHg (Figure 4C). For instance, the first streamflow pulse in Year 1a produced the largest peak of FMeHg that we ascribe to the mobilization of labile FMeHg from the surficial ash layer and eroded soil by the first rainfall event initiating streamflow generation following the wildfire (Figure 4C), a phenomenon also widely observed in streams within unburned landscape upon extensive soil erosion.^48^

Cache Creek displayed DOC-filtered Hg dynamics similar to that observed in Cold Creek (SI Figures S4C, S4D, S5C, and S5D). Similar to TSS, we did not observe much difference in DOC levels among the three study periods, with only a slight decline in median DOC levels over time (Year 1a = 4.9 mg/L, Year 1b = 4.3 mg/L, and Year 2 = 4.0 mg/L) (Figure 4A). Median FTHg concentrations followed a similar trend to DOC (median FTHg in Year 1a = 9.0 ng/L, Year 1b = 5.7 ng/L, and Year 2 = 2.5 ng/L) (Figure 4B). Finally, FMeHg was relatively elevated in Years 1a and 1b compared to Year 2 when the streamflow was substantially higher, possibly reflecting dilution of MeHg by the much larger stream discharge (Figure 4C). In Year 2, one can find that both sites show FMeHg values mainly below the average values in these streams as inferred from other studies (*see* dashed lines and figure captions of Figure 4C).

As widely shown in other stream studies,^28,48,49^ DOC was also found to be a positive predictor of FTHg and FMeHg in our fire-impacted streams (SI Figures S4C, D and S5C, D). However, the relationships were not particularly strong, especially for FMeHg, which is due in part to the relatively low levels and, in many cases, FMeHg concentrations below the analytical detection limit (ca. 0.04 ng/L) (Figure 4C). For study periods showing significantly positive relationships between DOC and FTHg, we found that Cold Creek demonstrated an increasing slope value from Year 1a (0.356 ± 0.211) to Year 1b (0.836 ± 0.259) and Year 2 period (1.287 ± 0.169) (SI Figure S4C). This potentially implies a combination of fire- and increasing rainfall-induced changes in the pools of FTHg and/or DOM, as well as postfire changes in hydrologic flow paths (e.g., surface runoff versus groundwater flow paths) following reestablishment of vegetation (SI Figure S2).

These slope values were found to be much higher than those of median (0.25) and mean (0.30) values observed in different North American studies.^29^ The values in Year 1b and Year 2 were also higher than the THg/DOC (median 0.56) in a watershed (Sagehen Creek) within the region in northern California.^50^ In Year 1a within Cold Creek, even when we removed the three water samples with relatively high FTHg but intermediate DOC levels, the slope between FTHg and DOC was 0.247 (*data not shown*). In contrast, we did not find any change in the slope in Cache Creek from Year 1a (2.68 ± 0.28) to Year 1b (2.21 ± 0.67), and there was no significant relationship in Year 2 (SI Figure S5C). Comparatively, these slopes are much higher than those observed in other fluvial systems, such as a blackwater river in southeastern USA during a flooding event (0.051–0.167)^36^ and rivers in Minnesota with (0.059–0.172) and without (baseflow; 0.045–0.119) storm runoff events.^27^ This implies that the DOC/DOM in these wildfire-affected streams can carry higher levels of Hg(II) than those in other wetland-dominated streams. However, a forested watershed in the southeastern U.S. found no evidence of a burning effect on the slope of the DOC vs FTHg relationship (burned: 0.529 vs unburned: 0.557).^5^ Differences in DOC/DOM quantity/quality, pH, and other solutes make direct comparison with other studies somewhat tenuous.^51^

Overall, the postfire-impacted streams did not appear to appreciably affect DOC and FTHg. The DOC and FTHg levels also decreased to levels similar to those at the reference site within the two-year study period as the flushing (leaching/erosion) of the ash and top soil layers occurred rapidly and the vegetation rapidly reestablished following the wildfires.^40^ For FMeHg, there may be some initial streamwater samples with slightly higher FMeHg, but their concentrations returned to very low levels often near our detection limit (0.04 ng/L) in Year 2. This implies that fluvial transport of MeHg was not a major concern from wildfire-impacted landscapes, even though PMeHg may pose a slight risk. We posit that Hg deposited with the eroded ash and soil in downstream aquatic sediments may potentially undergo microbial methylation Hg(II) to form MeHg if the biogeochemical conditions (i.e., redox condition and organic matter) are favorable.^8,52^ Compared to soil, ash, despite its high proportion of recalcitrant Hg,^6^ also possesses a high surface area and porous structure^8^ which can enhance Hg adsorption, potentially reducing its immediate reactivity and bioavailability but also affecting its long-term mobility as environmental conditions evolve.^6,8^ Over time, as erosion transitions from being predominantly ash-based to soil-based, the characteristics of exported Hg and importantly their impacts on the environmental Hg biogeochemical dynamics may shift accordingly.

### Implications of Wildfire on Mercury Pollution

Studying the impacts of wildfires on Hg cycling can only rely on unpredicted opportunities from natural disasters and cannot be planned in advance in a replicated manner. This study explored a unique opportunity in northern California to investigate two wildfire-impacted watersheds in relatively close proximity, one with a large burned area (90%) and lower background Hg levels (Cold Creek Watershed), and another watershed with a low burned area (15%) having higher background Hg levels (Cache Creek Watershed). Along with the inclusion of a nonburned reference watershed (Mill Canyon Creek Watershed), we used these comparisons to provide novel insights into the magnitude, processes, and recovery of the natural Hg cycle in burned landscapes. In this work, we documented extreme levels of suspended particulate mobilization from severely burned landscapes (*see* daily fluxes after normalization to watershed area in SI Figure S6), which were responsible for supplying high levels of inorganic Hg(II) and MeHg to downstream environments, mostly in the particulate phase. This has important implications for direct MeHg bioaccumulation and biomagnification in downstream aquatic ecosystems. Since the majority of transported Hg was in the form of particulate inorganic Hg(II) or MeHg, instead of dissolved Hg(II) or MeHg (the most bioavailable form), the more important question is under what conditions this deposited Hg(II) could be microbially methylated to become highly toxic MeHg in downstream environments.^8^ Further, this study also demonstrated the importance of the relatively rapid recovery of these burned watersheds to reduce mobilization of suspended particulates and associated Hg even in subsequent years with much larger rainfall and runoff/erosion events. This rapid recovery demonstrates the resilience of these fire-adapted ecosystems to future wildfire disturbance. Notably, the rapid recovery in stream Hg levels in these burned watersheds does not preclude potential long-term impacts on downstream Hg biogeochemical cycling processes such as methylation and bioaccumulation. These processes may continue to affect aquatic ecosystems beyond the immediate changes in Hg concentrations, potentially influencing Hg dynamics, trophic transfer, and exposure risks within the food webs.^17^

## Supporting Information Available

Detailed description of the sample processing and chemical analyses; summary of chemical data on various solid-phase materials; photos of the initial flush in the field and water samples; comparison of unfiltered mercury between Cold Creek and the reference stream; site photos of Cold Creek Watershed over time; relationships between the percentages of particulate mercury and particulate mercury level; relationships between total suspended solid and particulate mercury; temporal variations of dissolved organic carbon, filtered total mercury; and filtered methylmercury in both Cold Creek and Cache Creek; as well as their interrelationships (PDF)
