# Supplementary material for: Dominance of Particulate Mercury in Stream Transport and Rapid Watershed Recovery from Wildfires in Northern California, USA
Source: Environ Sci Technol. 2024 Dec 4;58(50):22159–69. doi: 10.1021/acs.est.4c09364 (PMC11656692; doi:10.1021/acs.est.4c09364)
Supplement: Supplementary file 1 — es4c09364_si_001.pdf [file es4c09364_si_001.pdf]

Supporting Information (SI)

Dominance of particulate mercury in stream transport and rapid watershed recovery from wildfires in northern California, USA

Peijia Ku [a,b], Martin Tsz-Ki Tsui [a,c,d,e *], Habibullah Uzun [f,g], Huan Chen [g], Randy A. Dahlgren [h], Tham C. Hoang [i], Tanju Karanfil [g], Huan Zhong [j], Ai-Jun Miao [j], Ke Pan [k], James S. Coleman [a], Alex Tat-Shing Chow [d]

[a] *Department of Biology, University of North Carolina at Greensboro, Greensboro, North Carolina 27402, USA*
[b] *Environmental Sciences Division, Oak Ridge National Laboratory, Oak Ridge, Tennessee 37831, USA*
[c] *School of Life Sciences, The Chinese University of Hong Kong, Shatin, N.T., Hong Kong SAR, China*
[d] *Department of Earth and Environmental Sciences, The Chinese University of Hong Kong, Shatin, N.T., Hong Kong SAR, China*
[e] *State Key Laboratory of Marine Pollution, City University of Hong Kong, Kowloon Tong, Kowloon, Hong Kong SAR, China*
[f] *Department of Environmental Engineering, Istanbul Technical University, Istanbul 34467, Turkey*
[g] *Department of Environmental Engineering and Science, Clemson University, Clemson, South Carolina 29625, USA*
[h] *Department of Land, Air and Water Resources, University of California, Davis, California 95616, USA*
[i] *School of Fisheries, Aquaculture and Aquatic Sciences, Auburn University, Auburn, Alabama 36849, USA*
[j] *School of the Environment, State Key Laboratory of Pollution Control and Resource Reuse, Nanjing University, Nanjing 210023, China*
[k] *Shenzhen Key Laboratory of Marine Microbiome Engineering, Institute for Advanced Study, Shenzhen University, Shenzhen 518060, China*

[*] Corresponding author. ***M.T.-K. Tsui --*** Room G89 Science Centre, The Chinese University of Hong Kong, Shatin, N.T., Hong Kong SAR, China; Tel.: +852-3943-6123; E-mail: mtktsui@cuhk.edu.hk

Summary: 13 pages, 6 figures, 2 tables

Text S1 -- Streamwater collection

For each sampling event along the hydrographs (**Fig. 1** in main manuscript), we collected streamwater sample into a 500-mL acid cleaned Nalgene Teflon bottle for Hg analyses and into a 1-L acid cleaned amber borosilicate glass bottle for general water chemistry analyses. In selected stormflow events, we deployed a 2-L Teflon bottle to collect sufficient streamwater sample to isolate the suspended solids for direct chemical analyses. Before any sample preservation or processing, all samples were shipped on ice overnight to the analytical laboratories at UNC-Greensboro (for Hg) and Clemson University (for general water chemistry; *see* the details in Uzun et al., 2020).

Text S2 -- Sample processing

From each 500-mL Teflon bottle, we filtered half of the streamwater through a pre-baked glass fiber filter paper (Whatman GF/F; nominal pore size of 0.7 μm), and analyzed both the unfiltered and filtered portions for Hg following our established laboratory protocols (Tsui et al., 2020; Ulus et al., 2022). From the samples in 2-L Teflon bottles, we first stored the samples at 4°C to allow the suspended particulates to fully settle, then we carefully drained the supernatant. We froze the remaining water-sediment slurry at -20°C and finally lyophilized the slurry to isolate the suspended particulates. Suspended particulates were analyzed directly for total Hg (THg) and calcium (Ca) (*see below*). All ash and soil samples were sieved through a 2-mm acid-cleaned polypropylene mesh prior to chemical analyses, including THg and Ca (Ku et al., 2018).

Text S3 -- Mercury analyses

For total Hg (THg) measurements, unfiltered and filtered water samples were fully digested with an acidic mixture of potassium permanganate ($KMnO_4$) and potassium persulfate ($K_2S_2O_8$) at 60°C overnight (Woerndle et al., 2018). Digested samples were neutralized by the addition of 30% hydroxylamine hydrochloride. Subsequently, an aliquot of sample (~50 to 120 mL) was analyzed for THg. All solid samples were digested by aqua regia following Ku et al. (2018). Digested reagent blanks and standard reference materials (SRMs) (National Research Council of Canada MESS-3 Marine Sediment) were included for quality assurance. THg in the reagent blank was consistently low (<1 ng/g) while the measured THg in MESS-3 was 94.8±2.7 ng/g (n=5), *vs.* a certified value of 91±9 ng/g (i.e., ~104% recovery). THg analysis was

conducted by the double amalgamation technique with Hg quantification by cold vapor atomic fluorescence spectrometry (CVAFS; Brooks Rand Model III, Seattle, WA, USA) (USEPA, 2002). For each analytical session, we developed a calibration curve (typically from 0 to 2 ng) with our primary working standard (NIST-3133 at 1 ng/mL) which was regularly checked against our secondary working standard (NIST-1641d at 1 ng/mL) (Ku et al., 2018; Tsui et al., 2020; Ulus et al., 2022). THg concentrations for streamwater samples were reported in ng Hg per liter (ng/L) for the particulate phase (as *PTHg*) or filtered phase (as *FTHg*); solid samples were reported in ng Hg per gram (ng/g) dry weight.

For MeHg measurements, unfiltered and filtered water samples were immediately preserved with 0.4% trace-metal grade hydrochloric acid (Parker and Bloom, 2005), and kept in the dark at 4°C. Prior to MeHg analysis, preserved water samples (~100 mL) were distilled to remove matrix interferences (Horvat et al., 1993). Distilled sample was then buffered with 200 µL of acetate buffer (pH ~4.9) in a glass bubbler, and then added with 50 µL of 1% ice-cold sodium tetraethylborate, and the mixture was agitated intermittently for 25 minutes. Alkyl mercury (Hg) species in the mixture were purged with a stream of Hg-free N_2 gas for 12 minutes into a Tenax TA trap. MeHg in the samples was quantified by CVAFS following gas chromatographic separation and pyrolysis (Horvat et al., 1993). The method detection limit (MDL) for MeHg in water samples was established at 0.02 ng/L, and we assigned a value of half the detection limit (i.e., 0.01 ng/L) for samples below the MDL for graphical presentation and calculations (Clarke, 1998). We used a MeHg calibration standard (1 ng/mL) obtained from CEBAM Analytical (Bothell, WA, USA), and we used this standard to develop a calibration curve (0 to 0.5 ng). The MeHg concentration in the working standard was regularly verified against our in-house THg standard solution (NIST-3133) using the method outlined by USEPA (1998).

We analyzed water samples for unfiltered THg (*UFTHg*), filtered THg (*FTHg*), unfiltered MeHg (*UFMeHg*), and filtered MeHg (*FMeHg*). We calculated the following parameters from these measured values: (i) particulate THg (*PTHg*, in ng Hg/L) = UFTHg – FTHg; and (ii) particulate MeHg (*PMeHg*, in ng Hg/L) = UFMeHg – FMeHg.

Text S4 -- Analyses of other chemical parameters

Streamwater from the 1-L amber glass bottle was filtered through a pre-filter (Whatman 934-AH), ~0.7-µm pore size) followed by a Supor®, PES 0.45-µm membrane, which was then analyzed for general water chemistry parameters (summarized in Table S1), but relevant to this study we mainly focused on TSS, DOC, and $SUVA_{254}$ (Uzun et al. (2020).

Solid particulate samples were dried and digested with aqua regia, and then diluted with ultrapure water, filtered before analysis for Ca content using inductively coupled plasma–mass spectrometry (Perkin Elmer; NeXion 300S).

Table S1. Summary of water quality analyses in this study. For details, please refer to Uzun et al. (2020).

**Parameter**	**Method**	**Equipment**	**Detection limit or precision**
Dissolved organic carbon	Standard Methods: 5310B	Shimadzu TOC-VCSH	0.1 mg/L
Total dissolved nitrogen	High Temp. Combustion	Shimadzu TNM-1	0.1 mg/L
UV absorbance	Standard Methods: 5910	Varian Cary 50 Bio UV-VIS Photometer	±0.004
pH	Standard Methods: 4500-H^+	VWR pH Meter Symphony B10P	±0.01

Table S2 A summary of total mercury (THg) and calcium (Ca) analyzed from the suspended particulates in Cold Creek (impacted by Wragg Fire) in Year 1a and 1b periods, black and white ash from Wragg Fire, and surface soils collected from burned and unburned zones of the Wragg Fire. It should be noted that data for wildfire ash have been previously reported in Ku et al. (2018). ND=no data.

Sample type	Sample information	THg (ng/g)	Ca (mg/g)
Surface soils	Unburned	48.3	13.3
	Unburned	31.5	9.2
	Unburned	35.7	10.9
	Unburned	75.8	16.6
	Unburned	18.9	14.2
	Unburned	45.1	15.6
	Under black ash 1	54.1	10.9
	Under black ash 2	111.7	12.3
	Under black ash 3	50.4	11.8
	Under black ash 4	35.5	9.8
	Under black ash 5	25.6	9.5
	Under white ash 1	40.7	16.4
	Under white ash 2	29.8	17.4
	Under white ash 3	8.4	12.4
	Under white ash 4	60.1	26.7
	Under white ash 5	5.9	14.5
Wildfire ash	Black ash 1	7.9	96.5
	Black ash 2	18.1	64.0
	Black ash 3	12.2	61.0
	Black ash 4	10.6	92.9
	Black ash 5	10.7	60.2
	White ash 1	9.2	249.3
	White ash 2	16.4	289.7
	White ash 3	14.8	262.0
	White ash 4	124.6	301.3
	White ash 5	8.8	280.1
Suspended particulates	5/1/2016 (10:30am)	ND	32.9
	5/1/2016 (2:10pm)	49.0	58.5
	6/1/2016	69.0	31.4
	7/1/2016	40.2	113.7
	18/1/2016 (7:40am)	40.1	27.3
	18/1/2016 (11:00am)	41.6	27.7
	19/1/2016	37.2	19.5
	5/3/2016	49.7	22.5
	13/3/2016	47.5	26.5

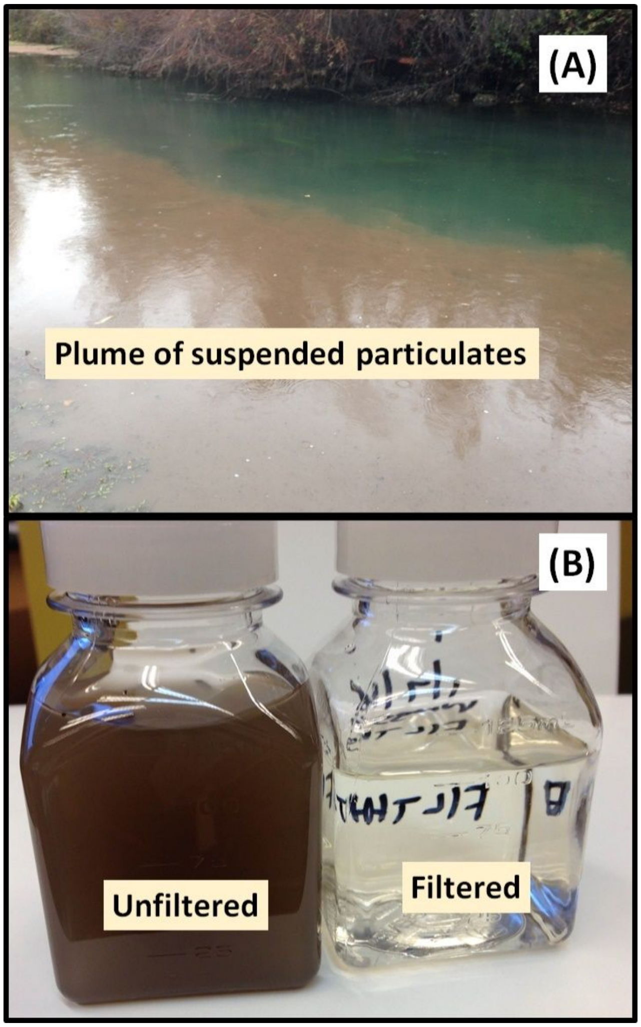

Figure S1. (A) Photo showing the first plume of suspended particulates in Cold Creek on 5 January, 2016 (photo credit: R. Dahlgren) at the confluence with Putah Creek, and **(B)** photo showing the difference between unfiltered and filtered streamwater samples collected from Cold Creek on 6 January, 2016 (photo credit: M. Tsui) with total suspended solids (TSS) level of 10,301 mg/L; the slight yellow tint in the filtered sample was due to a dissolved organic carbon (DOC) concentration of 12.0 mg/L.

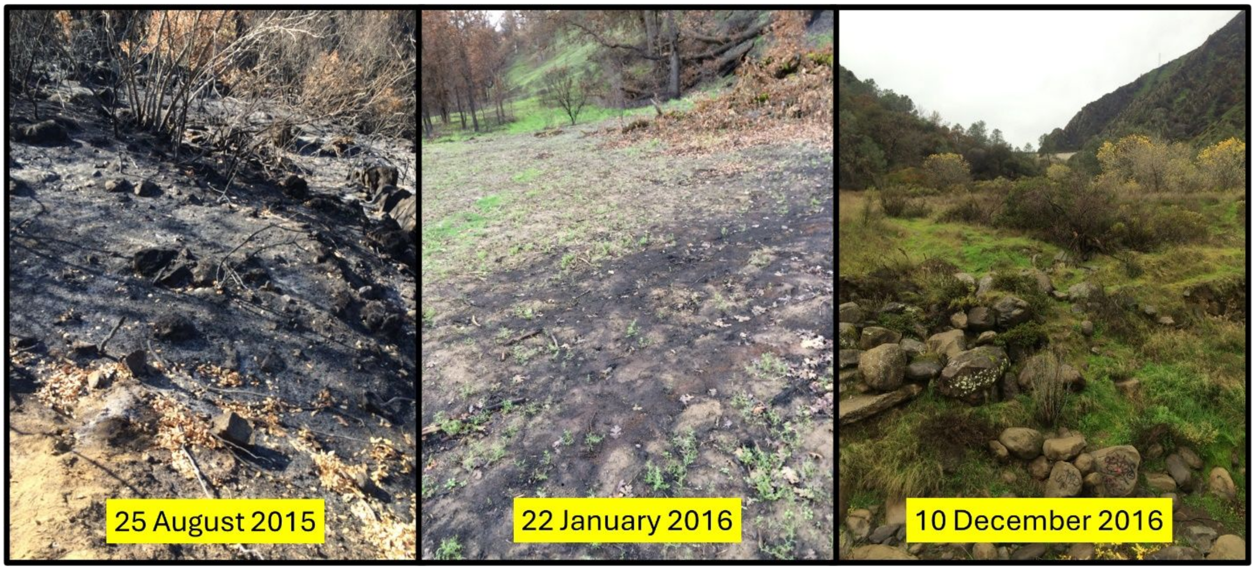

Figure S2. Field photos showing vegetation regrowth in part of the Cold Creek Watershed our team visited on 25 August 2015 immediately after the summer wildfire, 22 January 2016 after a few initial rainfall events in Year 1a, and 10 December 2016 at the onset of the wet season in Year 2 (photo credit: A. Chow).

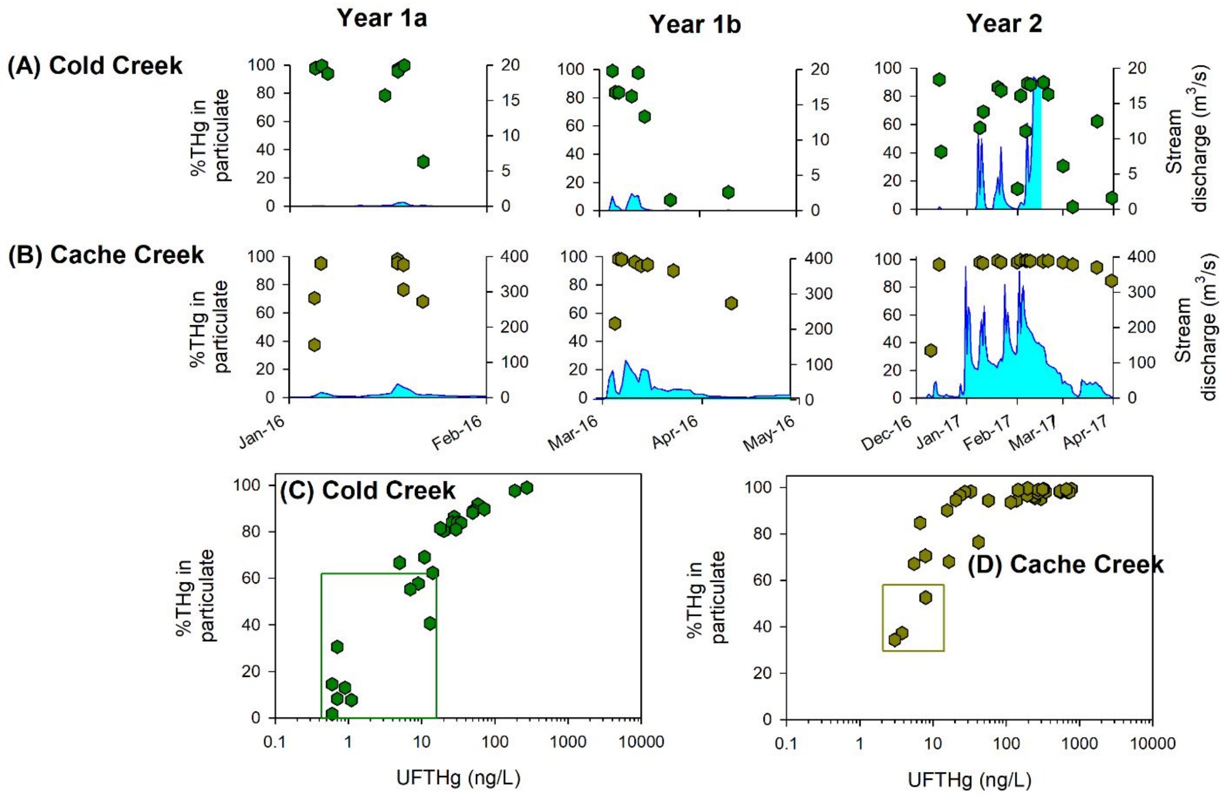

Figure S3. Temporal variations of streamwater percentage of total mercury (THg) associated with suspended particulates in **(A)** Cold Creek (impacted by Wragg Fire) and **(B)** Cache Creek (impacted by Rocky Fire) in Year 1a, Year 1b, and Year 2. The percentage of THg associated with suspended particulates as a function of unfiltered THg concentrations in streamwater in **(C)** Cold Creek and **(D)** Cache Creek. Boxes in (C) and (D) indicate the particulate samples with less than 60% of particulate THg.

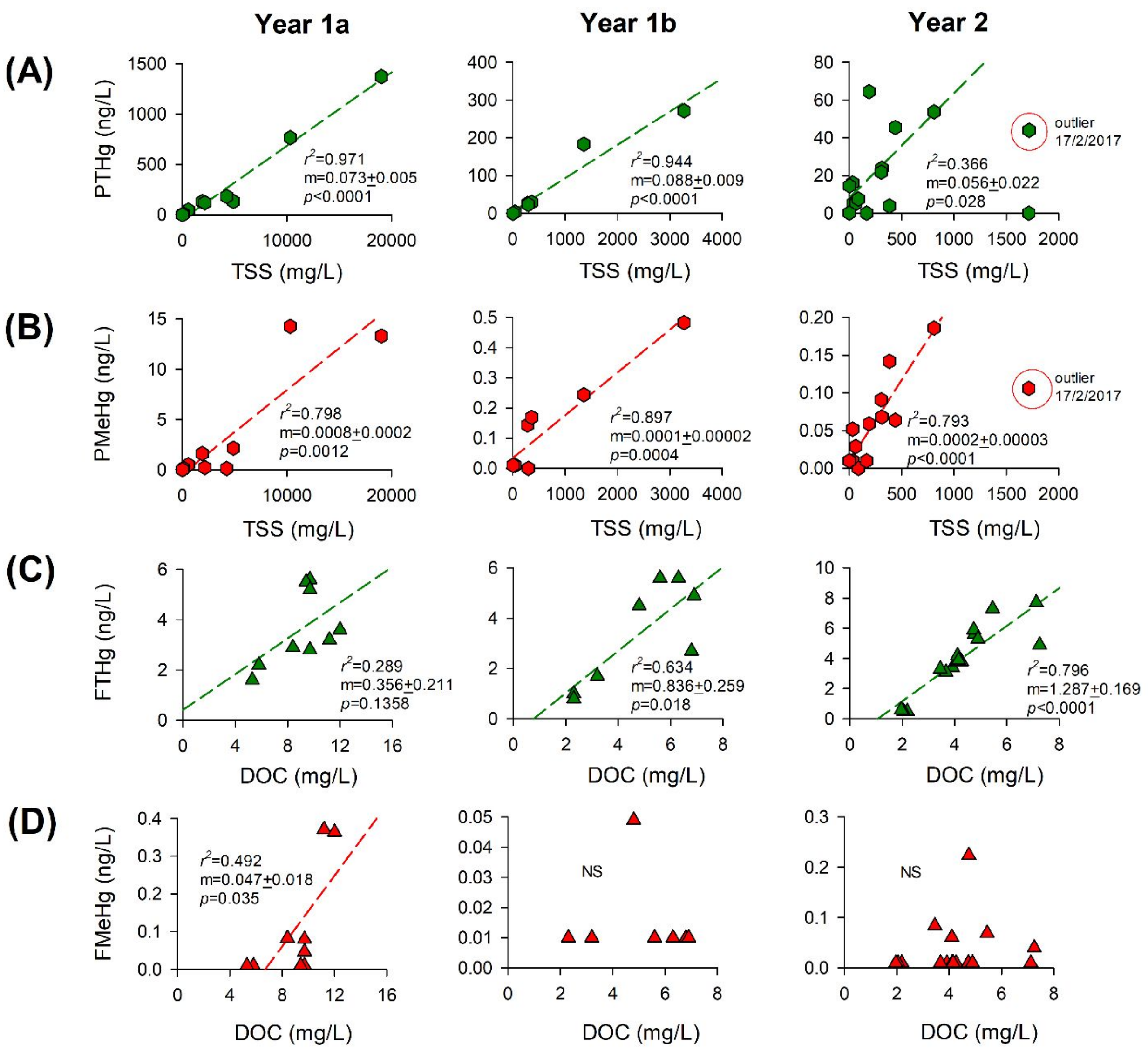

Figure S4. Relationships between ambient water quality parameters and mercury levels in streamwater at Cold Creek (impacted by Wragg Fire) during three different study periods (Year 1a, Year 1b, and Year 2). **(A)** TSS and PTHg, **(B)** TSS and PMeHg, **(C)** DOC and FTHg, and **(D)** DOC and FMeHg. An explanation for the outlier samples in Year 2 is provided in the main text. NS means no statistically significant relationship (p>0.05).

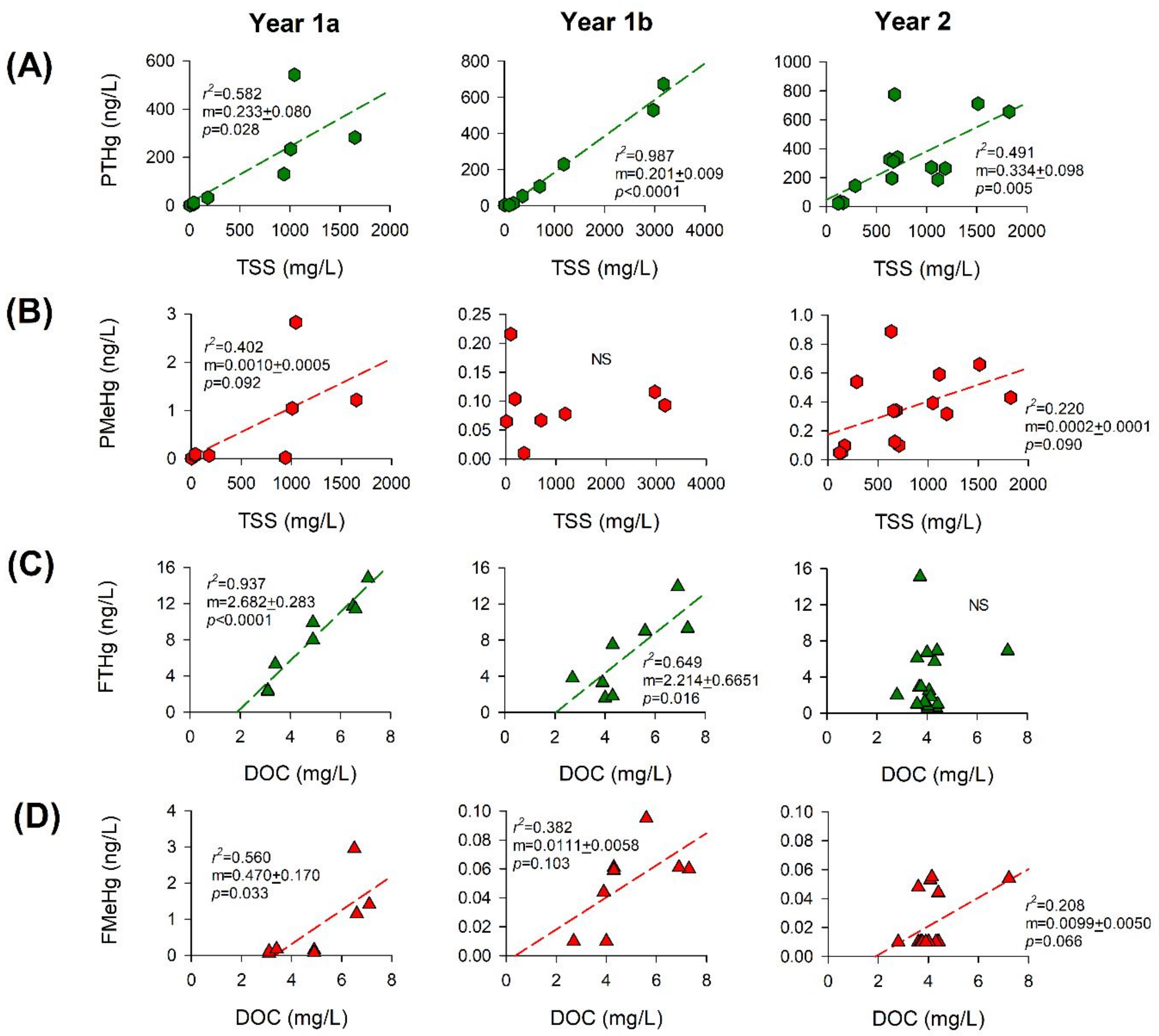

Figure S5. Relationships between ambient water quality parameters and mercury levels in streamwater at Cache Creek (impacted by Rocky Fire) during three different study periods (Year 1a, Year 1b, and Year 2). **(A)** TSS and PTHg, **(B)** TSS and PMeHg, **(C)** DOC and FTHg, and **(D)** DOC and FMeHg. An explanation for the outlier samples in Year 2 is provided in the main text. NS means no statistically significant relationship (p>0.05).

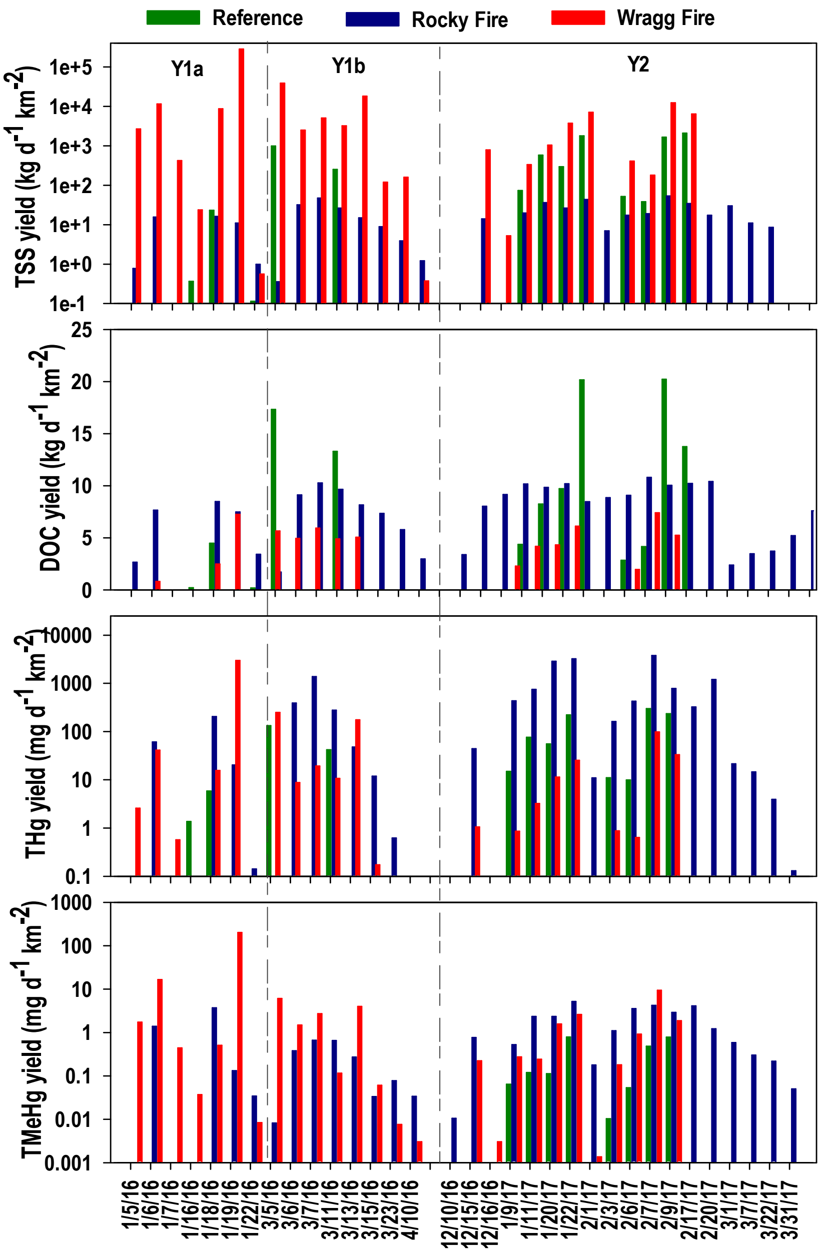

Figure S6. Daily yield comparisons of TSS, DOC, (unfiltered) THg, (unfiltered) TMeHg in Mill Canyon Creek (i.e., Reference), Cache Creek, and Cold Creek in the two post-fire years. The dashed line separated the periods of Year 1a (Y1a), Year 1b (Y1b), and Year 2 (Y2).

References for Supporting Information

Clarke, J. U. Evaluation of censored data methods to allow statistical comparisons among very small samples with below detection limit observations. *Environ. Sci. Technol.* **1998**, *32*, 177-183. https://doi.org/10.1021/es970521v

Horvat, M.; Bloom, N. S.; Liang, L. Comparison of distillation with other current isolation methods for the determination of methyl mercury compounds in low level environmental samples: Part 1. Sediments. *Anal. Chim. Acta* **1993**, *281*, 135-152. https://doi.org/10.1016/0003-2670(93)85348-N

Ku, P.; Tsui, M. T. K.; Nie, X.; Chen, H.; Hoang, T. C.; Blum, J. D.; Dahlgren, R. A.; Chow, A. T. Origin, reactivity, and bioavailability of mercury in wildfire ash. *Environ. Sci. Technol.* **2018**, *52*, 14149-14157. https://doi.org/10.1021/acs.est.8b03729

Parker, J. L.; Bloom, N. S. Preservation and storage techniques for low-level aqueous mercury speciation. *Sci. Total Environ.* **2005**, *337*, 253-263. https://doi.org/10.1016/j.scitotenv.2004.07.006

Tsui, M. T. K.; Uzun, H.; Ruecker, A.; Majidzadeh, H.; Ulus, Y.; Zhang, H.; Bao, S.; Blum, J. D.; Karanfil, T.; Chow, A. T. Concentration and isotopic composition of mercury in a blackwater river affected by extreme flooding events. *Limnol. Oceanogr.* **2020**, *65*, 2158-2169. https://doi.org/10.1002/lno.11445

Ulus, Y.; Tsui, M. T. K.; Sakar, A.; Nyarko, P.; Aitmbarek, N. B.; Ardón, M.; Chow, A. T. Declines of methylmercury along a salinity gradient in a low-lying coastal wetland ecosystem at South Carolina, USA. *Chemosphere* **2022**, *308*, 136310. https://doi.org/10.1016/j.chemosphere.2022.136310

USEPA. Method 1630, Methyl Mercury in Water by Distillation, Aqueous Ethylation, Purge and Trap, and Cold Vapor Atomic Fluorescence Spectrometry. Office of Water. U.S. Environmental Protection Agency. **1998.** Washington, D.C.

USEPA. Method 1631, Revision E: Mercury in Water by Oxidation, Purge and Trap, and Cold Vapor Atomic Fluorescence Spectrometry. Office of Water. EPA-821-R-02-019. **2002.** Washington, D.C.

Uzun, H.; Dahlgren, R. A.; Olivares, C.; Erdem, C. U.; Karanfil, T.; Chow, A. T. Two years of post-wildfire impacts on dissolved organic matter, nitrogen, and precursors of disinfection by-products in California stream waters. *Water Res.* **2020**, *181*, 115891. https://doi.org/10.1016/j.watres.2020.115891

Woerndle, G. E.; Tsui, M. T. K.; Sebestyen, S. D.; Blum, J. D.; Nie, X.; Kolka, R. K. New insights on ecosystem mercury cycling revealed by stable isotopes of mercury in water flowing from a headwater peatland catchment. *Environ. Sci. Technol.* **2018**, *52*, 1854-1861. https://doi.org/10.1021/acs.est.7b04449
